# Admission Pattern of Gastrointestinal Cancer for 2020–2023 From a Single Tertiary-Care Hospital in Pune, Western Maharashtra

**DOI:** 10.7759/cureus.67248

**Published:** 2024-08-19

**Authors:** Mahesh Thombare, Nikhil Jillawar, Vidyachandra Gandhi, Aditya Kulkarni, Ajay Vane, Veena Joshi, Madhura Deshmukh

**Affiliations:** 1 Surgical Gastroenterology, Dr. D. Y. Patil Medical College, Hospital and Research Centre, Dr. D. Y. Patil Vidyapeeth (Deemed to be University), Pune, IND; 2 Medical Research, Dr. D. Y. Patil Medical College, Hospital and Research Centre, Dr. D. Y. Patil Vidyapeeth (Deemed to be University), Pune, IND; 3 Central Research Facility, Dr. D. Y. Patil Medical College, Hospital and Research Centre, Dr. D. Y. Patil Vidyapeeth (Deemed to be University), Pune, IND

**Keywords:** retrospective analysis, diagnosis, demographics, patterns, gastrointestinal cancers

## Abstract

The prevalence of gastrointestinal (GI) cancer is increasing across diverse regions of India, demanding further investigation at the state level. In response, a new department of surgical gastroenterology was started at a tertiary-care hospital in Pune, Western Maharashtra, in 2019. The objective of this study was to explore the pattern of admissions in terms of demographics and types of GI cancers over the last four years (i.e., 2020-2023).

Retrospective admissions data were collected from hospital records for 2020-2023. A total of 2294 patients were treated at the outpatient department (OPD), and 135 patients were admitted to the inpatient department (IPD). The data comprised OPD/IPD admissions, age, gender, diagnosis, and length of stay (LoS). In addition to basic statistical reporting, t-tests were used to explore differences among the study variables.

Out of 135 GI cancer patients, 57% were male. The mean age of inpatients per year ranged from 53 to 60 years, with an average age of 56.35 ± 10.14 years. The average LoS was 12.31 ± 9.39 days. From 2020 to 2023, the number of admissions increased from 5 to 57. The increase was more pronounced in men than women (57% vs. 43%, respectively). Furthermore, increased admission of younger patients was observed, and the average LoS decreased from 17 to 11 days from 2020 to 2023, respectively. A statistically significant difference in LoS (p = 0.023) was observed based on gender, where LoS was longer for women than for men on average (13.5 ± 10.8 vs. 9.46 ± 8.28, respectively).

As GI cancer incidence is predicted to continue to increase in India, these new estimates will help to plan cancer prevention and control through intervention via* *early detection and management.

## Introduction

The burden of cancer is increasing both in India and globally. According to the Indian Council of Medical Research (ICMR)-National Centre for Disease Informatics and Research, it is likely that the incidence of cancer cases in India will increase from 1.46 million in 2022 to 1.57 million in 2025 [1]. Gastrointestinal (GI) cancers represent a quarter of all cancer cases, including esophageal, stomach, hepatobiliary, pancreatic, and colorectal cancers. The prevalence of these cancers is continuously increasing globally [2-4].

GI cancers include different varieties and distributions worldwide [5]. As GI cancers comprise many different cancer types, it has a very high burden in terms of incidence and adverse outcomes globally, including in India [6]. The prevalence and incidence of GI cancers vary across different geographic locations. In Arnold’s study on the global burden of five major types of GI cancer, analysis of the Global Cancer Observatory (GLOBOCAN) database indicated that esophageal, gastric, and liver cancers were more common in Asia than in other parts of the world in 2018 [3].

Shakuntala et al. conducted a study on the descriptive epidemiology of GI cancers and found that the occurrence of GI cancers was more common among men (60.5%) than among women (39.5%). The age-adjusted incidence rate (AAR) of GI cancer was highest in India’s northeast region, with 126.9 per 100,000 men in the Aizwal district and 75.9 per 100,000 women in Papum Pare. Furthermore, the most prevalent cancer among men and women was cancer of the esophagus at 28.2% and 25.7% of cases, respectively [7].

This retrospective study analyzed inpatient admissions data from the department of surgical gastroenterology of a tertiary-care hospital in Western Maharashtra. The objective of the study was to understand the diagnosis of GI cancers and investigate trends regarding the number of admissions, age, and length of stay (LoS) of GI patients from 2020 to 2023.

## Materials and methods

The study was conducted at the newly established (i.e., 2019) department of surgical gastroenterology at Dr. D. Y. Patil Hospital in Western Maharashtra, India; it included patients diagnosed with GI cancers of both luminal and solid organ types who were admitted to the hospital for the treatment. As a policy, the department does not manage oral-pharyngeal cancers or salivary gland cancers.

The department maintains registries of inpatient and outpatient data. From 2020 to 2023, there were 1603 new visits and 722 readmissions in the outpatient department (OPD). The data for this study comprised 135 inpatients who were hospitalized for treatment. Multiple admissions of the same patient were counted only once.

The inpatient department (IPD) data were retrospectively analyzed in terms of number of admissions over the study period by month and year, as well as demographic characteristics such as age, gender, diagnosis, and LoS to investigate admission patterns. 

Ethical approval

The Institutional Ethical Subcommittee of Dr. D. .Y. Patil Medical College, Hospital and Research Centre approved the study (Ref. No.: I.E.S.C./W/32/2024).

Statistical analysis

Data were entered in MS Excel (Microsoft Corporation, Redmond, Washington, United States) and exported to IBM SPSS Statistics for Windows, Version 27 (Released 2020; IBM Corp., Armonk, New York, United States) to determine various trends (number of patients, age, and LoS), differences, and associations among the study variables. Independent t-tests were used to compare gender differences in terms of age and LoS.

## Results

Records of 135 patients (57% males and 43% females) admitted to the study center from 2020 to 2023 for management of GI cancer were analyzed. The mean age of inpatients per year ranged from 53 to 60 years, with an average age of 56.35 ± 10.14 years. The average LoS was 12.31 ± 9.39 days (Table 1).

**Table 1:** Demographic characteristics of admitted patients (n = 135) LoS: Length of stay; SD: standard deviation Data presented as numbers or means (±SDs)

Year	Number of patients	Age (years), mean (±SD)	LoS (days), mean (±SD)
2020	5	59.80 (±7.0)	10.76 (± 9.2)
2021	28	55.30 (±11.2)	17.00 (± 9.2)
2022	45	57.37 (±15.1)	9.14 ( ± 8.4)
2023	57	52.94 (±7.0)	12.34 (± 10.6)

**Figure 1:**
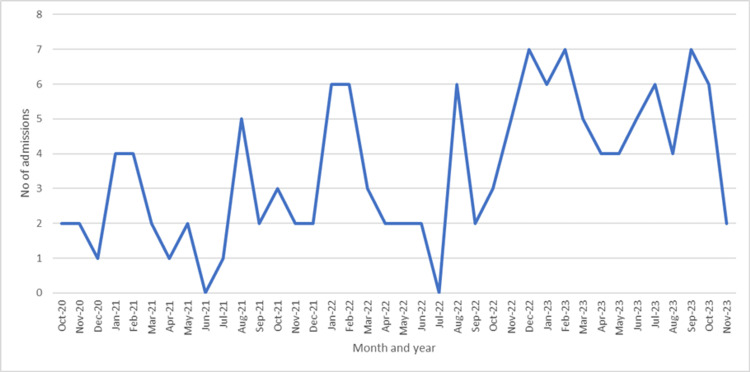
Monthly number of admissions of gastrointestinal cancer patients (n = 135) from October 2020 to November 2023

Month-wise admissions to the IPD of surgical gastroenterology are shown in Figure 1.

**Figure 2:**
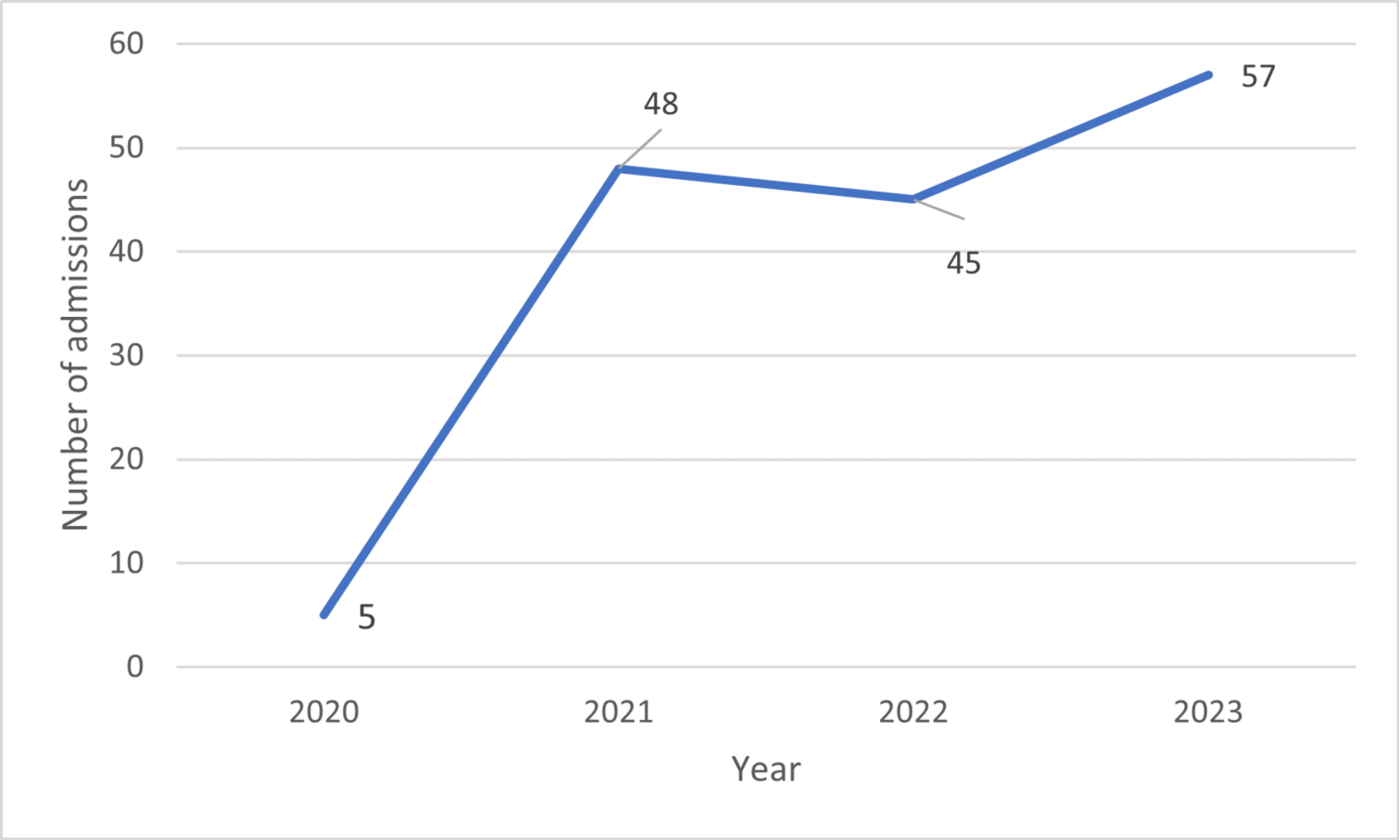
Number of yearly admissions from 2020 to 2023

The number of admissions increased from 5 to 57 from the year 2020 to 2023, respectively, and this increase was observed more in men than in women (Table 1 and Figure 2).

In 2023, more men (n = 38) were admitted for GI cancer than women, whereas more women (n = 23) were admitted in 2022 (Table 2).

**Table 2:** Yearly gender distribution (n = 135) Data are presented as numbers or numbers (%)

Gender	2020	2021	2022	2023	Total
Male	1	16	22	38	77 (57%)
Female	4	12	23	19	58 (43%)
Total	5	28	45	57	135 (100%)

Incidence of GI cancer was higher in men than in women at 57% vs. 43%, respectively, with no statistically significant differences in age observed between both groups.

A statistically significant difference in LoS (p = 0.023) was observed based on gender, where LoS was longer on average for women than for men(13.5 ± 10.8 vs. 9.46 ± 8.2) (Table 3).

**Table 3:** Distribution of length of hospital stay across genders (n = 135) LoS: Length of stay Data are presented as means (±SDs). p-value obtained by t-test

Gender	Number of patients	LoS (days), mean (±SD)	p-value
Male	76	9.46 (±8.2)	0.023
Female	58	13.50 (±10.8)	

**Figure 3:**
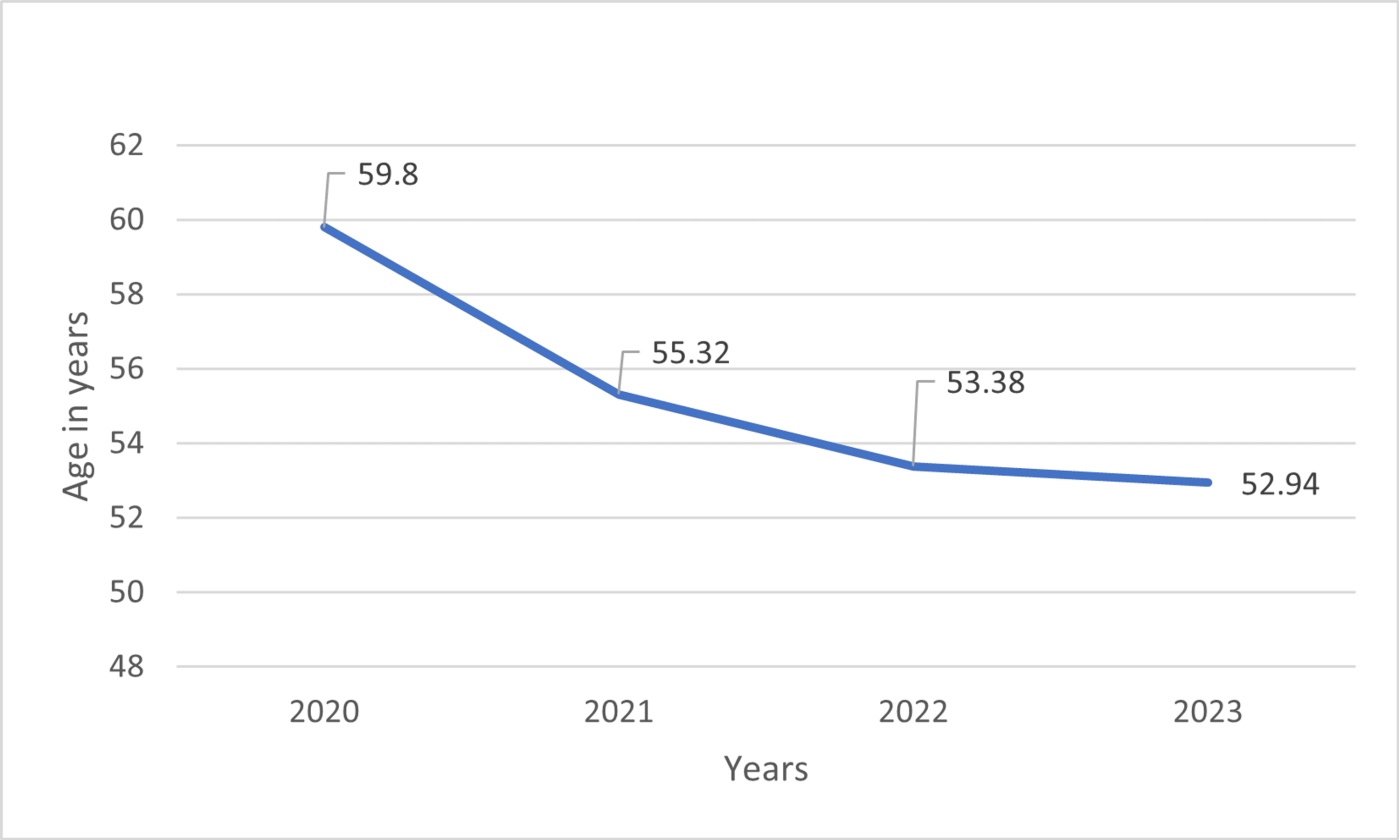
Average age at which patients were admitted by year

The average age for GI cancer patients admitted from 2020 to 2023 is shown in Figure 3. Patient admissions increased over the four years of the study period. During this time, the average age at which patients were admitted decreased from 60 to 53 years from 2020 to 2023, respectively, indicating that younger patients were being admitted.

**Figure 4:**
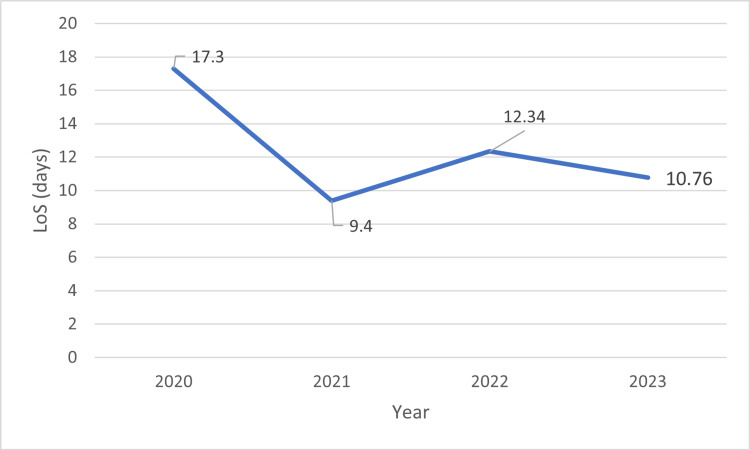
Average LoS for GI cancer patients admitted from 2020 to 2023

The average LoS for GI cancer patients admitted from 2020 to 2023 is shown in Figure 4.

As seen in Figure 4, LoS decreased from 17.3 to 10.76 days from 2020 to 2023, respectively. Our results showed that among the 135 patients admitted to our single tertiary-care center during 2020-2023, the most common GI cancers observed were colorectal cancer (n = 15; 11%), esophageal cancer (n = 11;7%), liver cancer, and gallbladder cancer (n = 8; 6%). The average age of 15 cases of colorectal cancer was 56.8 ± 8.74 years and LoS was 12.67 ± 11.75 days, and there were 53% males. There were a few cases of GI stromal tumors (GISTs), pancreatic cancer, and periampullary cancer, for which the incidences in India are discussed below.

## Discussion

Hospital admissions patterns aid in understanding the utilization of health care services and illustrating the disease burden prevailing in communities served. In this study, we attempted to determine the current pattern of admissions of GI cancers in our four-year-old department of surgical gastroenterology. The main GI cancers observed were colorectal, esophageal, liver, and gallbladder cancers. The number of inpatient admissions increased from five to 57 (a factor of 10.4) over the four years of the study.

Our study showed that those admitted for GI cancer were mainly between 50 and 60 years of age. The number of admissions increased from 2020 to 2023, which may be because of word-of-mouth publicity and the maturity of the department. Over the same period, the average age of those admitted decreased from 60 to 53 years, respectively, indicating that more people developed GI cancer at an earlier age which is based on our hospital data. Furthermore, the average LoS decreased from 17 days to 11 days, respectively, which may be because of more aggressive management (as per the treating physicians' opinion from our hospital) with enhanced recovery measures taken.

The incidence of GI cancer varies across different countries. As stated in a study by Arnold, overall GI cancer incidence rates will continue to fall in the future in high-incidence countries such as Japan and low-incidence countries such as Australia [3]. The same study did not mention India, but according to the online publication Health World, India, has a comparatively high incidence of GI cancer compared to Western countries, and prevalence has increased in the last 10 years [8].

Pancreatic and periampullary cancer are rare in India. Gaidhani et al. stated in their article that pancreatic cancer ranked 24th with 10,860 (1.03%) new cases [9]. According to Qayoom et al., periampullary carcinoma contributed to 0.5%-2% of gastrointestinal malignancies [10]. Fewer patients in our study reported pancreatic or periampullary carcinoma cancer.

In a study by Sharma et al., except for gall bladder cancer, all GI cancers exhibited a predominance in men [11], as observed in our study. In India, esophageal cancer is the fourth most common cause of cancer-related deaths [12]. Our study also showed a higher number of esophageal cancers compared to other cancer types. In India, the annual incidence of hepatocellular carcinoma (HCC) in cirrhosis is 16%. However, unpublished data from various tertiary-care centers suggest that the incidence of HCC is increasing in India [13]. GIST, the most common mesenchymal tumor found in the alimentary tract, may arise from any part of the GI tract and account for <1% of all GI cancers [14], which is in line with the results of our study.

A review article reported gender-specific and age-adjusted colorectal cancer incidence after conducting a pooled analysis from 27 population-based cancer registries in India. The results showed the annual incidence rates for colon cancer and rectal cancer in men were 5.36 and 5.17 out of 100,000 population, respectively, and that of colon cancer in women was 4.3 out of 100,000 population [15]. During the year 2010, there were 14,000 cancer deaths in India due to liver cancer, with an age-standardized cancer mortality rate of 6.8 (5.4-8.1) per 100,000 cases [16].

This retrospective study had some limitations. Surgical gastroenterology is a very young department at Dr. D. Y. Patil Vidyapeeth Hospital, and this was a single-center study, with only a small sample of admission data. Therefore, a direct comparison of incidence with other studies was not possible. Furthermore, the retrospective data did not capture information related to socioeconomic status, comorbidities, and other information related to clinical parameters. Additionally the monthly of admission were very small (<5); hence, we we reported the average of yearly admissions.

The results of the present study were consistent with those from other studies conducted in India. The present hospital admission patterns allowed us to understand the utilization and application of healthcare services, as they indicated the demographic-related GI cancer disease burden prevailing in the community.

## Conclusions

This study attempted to determine admission trends for GI cancer at a tertiary hospital in Pune, Western Maharashtra, India. The new estimates will help to plan cancer prevention and control through intervention via early detection and management.

Our study emphasizes that younger patients are being hospitalized for GI cancer. To address this challenge, age- and gender-specific programs are crucial, which should focus on risk factor modification, cancer awareness, and promoting early detection through accessible screening, and timely diagnosis and care.
